# Social Network Clustering Analysis for Detection of Associated Genetic Co-Mutations in Patients with Actionable Driver Mutations in NSCLC

**DOI:** 10.3390/life16071071

**Published:** 2026-06-26

**Authors:** Abed Agbarya, Haitham Nasrallah, Kamel Mhameed, Edmond Sabo, Walid Shalata, Esti Liani, Salam Mazareb, Mohammad Sheikh-Ahmad, Leonard Saiegh, Dejan Radonjic, Viktor Sebek, Dan Levy-Faber

**Affiliations:** 1 The Ruth and Bruce Rappaport Faculty of Medicine, Technion-Israel Institute of Technology, Haifa 3109601, Israel; danfaber1@hotmail.com; 2 Department of Oncology, Bnai-Zion Medical Center, Haifa 3339419, Israel; 3 The Joseph Fishman Oncology Center, Rambam Medical Center, Haifa 3525409, Israel; h_nasrallah@rmc.gov.il (H.N.); k_mhameed@rmc.gov.il (K.M.); 4 Pathology Institute, Lady Davis Carmel Medical Center, Haifa 3436212, Israel; edmondsa@clalit.org.il (E.S.); esti.liani@clalit.org.il (E.L.); mazareb_salam@clalit.org.il (S.M.); 5 The Legacy Heritage Cancer Center, Dr. Larry Norton Institute, Soroka Medical Center, Beer Sheva 8457108, Israel; walid.shalata@gmail.com; 6 Faculty of Health Sciences, Ben Gurion University of the Negev, Beer Sheva 8410501, Israel; 7 Institute of Endocrinology, Bnai-Zion Medical Center, Haifa 3339419, Israel; mohammad.ahmad@b-zion.org.il (M.S.-A.); leonard.saiegh@gmail.com (L.S.); 8 Faculty of Medicine and Dentistry, Palacky University, Hnevotinska Street 3, 77515 Olomouc, Czech Republic; dejan_radonjic@msn.com; 9 Department of Pharmacology, Faculty of Medicine and Dentistry, Palacky University, Hnevotinska Street 3, 77515 Olomouc, Czech Republic; sebek.viktor@gmail.com; 10 Department of Cardiothoracic Surgery, Lady Davis Carmel Medical Center, Haifa 3436212, Israel

**Keywords:** non-small cell lung cancer (NSCLC), actionable driver mutations, social network analysis (SNA), co-mutations, programmed death-ligand (PD-L1), tumor mutation burden (TMB)

## Abstract

Non-small cell lung cancer (NSCLC) exhibits genomic heterogeneity that affects tumor immunogenicity and PD-L1 expression. Patient clustering based on shared mutational profiles using social network analysis (SNA) has been narrowly explored. The study aimed to identify subgroups of NSCLC patients with similar somatic mutation profiles using network-based modularity clustering, and to compare these groups with respect to PD-L1 expression, Tumor mutation burden (TMB), and clinical variables. Data of patients with stage 4 (metastatic) NSCLC, whose tumor tissue samples were collected between 2022 and 2024, were analyzed. This retrospective study included NSCLC patients harboring actionable driver mutations in genes such as *EGFR*, *KRAS*, *ALK*, *BRAF*, *MET*. A social network of 129 patients was constructed. Two distinct genomic clusters were identified. Cluster 2 (*n* = 55) showed a higher prevalence of *KRAS*, *TP53*, *BRAF*, *STK11* and additional mutations, while cluster 1 (*n* = 74) displayed a limited number of driver mutations. Cluster 2 had significantly higher PD-L1 expression (29.8% vs. 13.7%, *p* = 0.001) and higher TMB (7.8 vs. 5.8, *p* = 0.021). In multivariate logistic regression, both PD-L1 and TMB were associated with cluster assignment (*p* < 0.05). Mutation-based SNA clustering delineated two biologically distinct subgroups of NSCLC patients. The highly mutated cluster displayed higher PD-L1 expression and TMB, a profile consistent with a more immunogenic phenotype. This method offers a novel integrative approach that requires prospective validation before clinical implementation.

## 1. Introduction

Non-small cell lung cancer (NSCLC) accounts for about 85% of all lung cancer cases and is considered the leading cause of cancer mortality worldwide, with approximately 1.35 million new cases and 1.18 million deaths each year [1]. About 70% of patients are diagnosed at an advanced stage (stage 4), with a five-year survival rate of less than 5% [2].

Cancer cells are known to harbor genetic alterations, some of which drive cancer progression and oncogenesis. Detection of the driver mutation is important for diagnosis, prognosis and planning therapy [3]. Advances in genomic testing have fundamentally changed the treatment approach to lung cancer [4]. Mutations in *EGFR*, alterations in *ALK*, *ROS1*, *BRAF*, *MET*, and *KRAS* serve as therapeutic targets and are identified in a cumulative rate of about 20–40% of patients [5]. Targeted therapies (tyrosine kinase inhibitors, TKIs) have led to improvements in lung cancer/tumor response and time to disease progression; however, resistance usually develops within a year [6].

Additional progress in lung cancer treatment has occurred thanks to immunotherapy, particularly the blockade of the programmed cell death protein-1/programmed death-ligand-1 (PD-1/PD-L1) axis [7]. About 25–30% of pulmonary tumors express high levels of PD-L1 (presented as tumor proportion score (TPS) ≥50%), and respond better to treatment with drugs such as pembrolizumab, nivolumab, and atezolizumab [8]. Studies such as KEYNOTE-024 demonstrated a significant improvement in survival with pembrolizumab compared to chemotherapy in NSCLC patients expressing high levels of PD-L1 [8].

In addition, a high tumor mutational burden (TMB) in lung cancer is a biomarker associated with a higher likelihood of response to immunotherapy [9]. However, tumors with mutations in *EGFR* or *ALK* tend to have a suppressed immune microenvironment and a lower response to immunotherapy [10], in contrast to mutations in *KRAS*, *MET*, and *BRAF*, which may be more immunogenic [11].

PD-L1 expression varies greatly among groups based on mutational profile [12]. For example, over 50% of tumors with an *EGFR* mutation express PD-L1 [13].

TA correlation was found between different mutations and the expression of PD-L1 in tumors, for example: mutations that increase PD-L1 expression are *TP53*, *PTEN LOSS*, *MYC*, *KRAS*, *BRAF*, *MET* (indirectly) [14]. Mutations that suppress PD-L1 expression are *EGFR*, *ALK*, *STK11*, *KEAP1*, *NFE2L2* [15,16,17,18,19]. Moreover, there are mutations with variable effects on PD-L1 expression such as *PIK3CA*, *ERBB2*, *ROS1*, *RET* [20,21].

Possible clinical implications arise from the correlations between mutations and PD-L1 expression, for example, tumors harboring *KRAS* and *TP53* mutations have high PD-L1 expression, implying an inflammatory microenvironment, thus they have a high likelihood of response to immune checkpoint inhibitor (ICI)-based therapies. Tumors harboring *KRAS* and *STK11* mutations or *KRAS* and *KEAP1* were found to have low PD-L1 expression (cold immune environment), thus poor response to immunotherapy. Tumors driven by *EGFR* or *ALK* sometimes express PD-L1 but usually without significant immune infiltration; therefore, they have a limited benefit from PD-1/PD-L1 inhibitors [20,21,22,23].

The standard methods of genetic analysis often focus on a single mutation or a single biomarker [24]. However, a systemic approach that integrates multi-dimensional genomic information allows a deeper understanding of the connections between patients. Social network analysis (SNA) offers an innovative methodology for identifying links between patients based on their somatic mutation profiles [25]. This approach enables the creation of groups of patients with genomic similarity—which may reveal subgroups with different biological and immune characteristics.

This approach is based on the pioneering work of Prof. Albert-László Barabási, who was among the first to apply the mathematics of social networks to biological systems. Barabási demonstrated how genes, proteins, and enzymes operate as part of a complex biological network, rather than as isolated individuals. These insights laid the foundation for the field of “Network Medicine”, which offers innovative ways to analyze diseases—and in this case, to study lung cancer [26].

**Figure 1:**
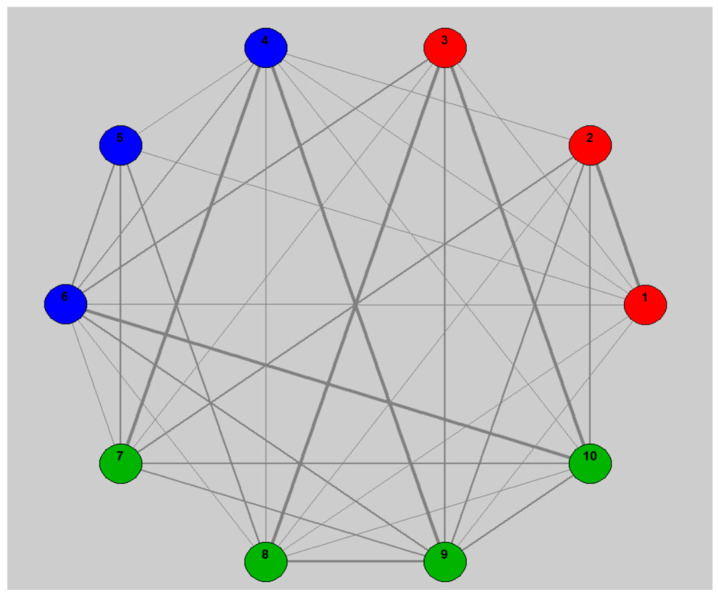
Illustration of a simulated example of a social network of patients linked by shared mutations. The color of the patients reflects the group, i.e., the shared cluster.

The current study aims to examine whether the aforementioned correlations hold up in reality among NSCLC stage 4 patients at the Carmel Medical Center (CMC). To this end, a method that constructs a social network of patients according to shared mutations (Figure 1) is proposed and based on this network performs clustering of patients into groups with shared mutations. The PD-L1 expression in the different tumors will be compared.

Research Hypothesis

Patients with metastatic NSCLC stage 4 lung cancer who are grouped according to shared somatic mutation profiles will show different PD-L1 expression profiles and different demographic characteristics, indicating variability in tumor immunogenicity and disease traits.

Research Objectives

1. To identify groups of patients with similar genomic profiles using social network and modularity analysis.
2. To compare these groups according to PD-L1 expression levels in order to characterize the immunological differences between them.
3. To examine differences between the groups in demographic and clinical variables (age, gender, smoking, histology).

## 2. Materials and Methods

### 2.1. Study Design and Population

This single-center non-interventional retrospective study included 129 patients, aged 18 and older, diagnosed with stage 4 NSCLC who underwent genetic sequencing (NGS) and PD-L1 testing by immunohistochemistry at the Institute of Pathology of Carmel Medical Center [27]. Another inclusion criterion was patients having medical records data with demographics, clinical information, and lung tumor biopsy samples collected between 2022 and 2024. Exclusion criteria were patients aged <18 years old, incomplete medical records information or missing information of NSCLC tumor sample sequencing or PD-L1 testing.

### 2.2. Data Collection

Data Collection was composed of a patient identifier (pseudonym), somatic mutation profile (gene level such as KRAS, TP53, BRAF, or STK11), TPS result for PD-L1, age, gender, and ethnic origin. PD-L1 expression was assessed by immunohistochemistry and recorded as the tumor proportion score (TPS), defined as the percentage of viable tumor cells showing partial or complete membranous staining. Tumor mutational burden (TMB) and the somatic mutation profile were derived from the same next-generation sequencing (NGS) assay performed on the tumor biopsy samples.

### 2.3. Ethics

The study was conducted in accordance with the Declaration of Helsinki, and approved by the Institutional Review Board Ethics Committee of Carmel Medical Center protocol code CMC-0073-24 on 23 September 2024. Patient consent was waived due to the retrospective nature of the study.

### 2.4. Construction of the Social Network

The social network was constructed by the following steps: First, each patient was defined as a node. Second, an edge was created between any two patients sharing at least one somatic mutation. Third, the edge weight was determined by the number of shared mutations, so that patients with more genomic similarity received a stronger connection. Fourth, a weighted undirected network was obtained that represented the genomic proximity between patients. Somatic alterations were considered at the gene level: multiple variants within the same gene were collapsed to a single gene-level call, and copy-number changes and variants of uncertain relevance were not included in the similarity matrix. Edge weight was defined solely as the count of genes shared between two patients, without weighting for the functional importance, pathway membership, or predicted impact of individual mutations; consequently, driver and passenger gene-level calls contributed equally to network connectivity. This deliberately simple formulation is acknowledged as a limitation in Section 4.

### 2.5. Louvain Algorithm and Modularity Measure

The Louvain algorithm is a fast hierarchical algorithm for detecting clusters in large networks [28]. It operates by maximizing modularity—the measure of link density within a cluster compared to links between clusters. High modularity means “natural” clusters where there is significant similarity among cluster members and few external links. The algorithm iteratively applies merging and splitting of clusters until the optimal partition is found. In this study, two distinct patient clusters were obtained.

### 2.6. Statistical Analysis

#### 2.6.1. Between-Group Comparisons

Continuous variables (age, PD-L1, TMB) were compared using a two-sample *t*-test (Student’s *t*-test). Categorical variables (gender, ethnicity) were compared using χ^2^ tests to examine between-group differences. A *p*-value of ≤0.05 was considered statistically significant. Owing to the limited number of pre-specified comparisons, *p*-values are reported without formal correction for multiple testing and are interpreted as exploratory. Analysis was performed using IBM SPSS version 26 (IBM, Armonk, NY, USA).

#### 2.6.2. Logistic Regression

A multivariate logistic regression was performed, in which the dependent variable was cluster membership (1 or 2), and the independent variables were: age, gender, ethnicity, PD-L1, and TMB.

The goal was to identify variables associated with patient assignment to the genomic cluster.

During the preparation of this manuscript/study, the authors used the AI tools ChatGPT virsion 4.5, deep research to help identify mutation patterns within algorithmic clusters. The authors have reviewed and edited the output and take full responsibility for the content of this publication. During manuscript preparation, no patient-level, identifiable, or case-level genomic data were entered into the AI system. All clustering, statistical analyses, and interpretation of genomic patterns were derived from the statistical software outputs and the underlying dataset.

## 3. Results

### 3.1. Social Network Clusters

**Figure 2:**
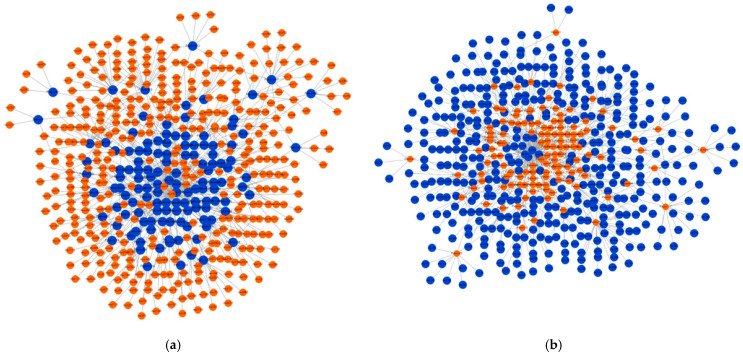
(**a**) A social network of patients (marked in blue), connected to each other through shared mutations (marked in orange). (**b**) A social network of mutations (marked in blue), connected to each other through shared patients (marked in orange).

Following the construction of the social network and the operation of the Louvain algorithm, the patients were divided into two clusters based on the mutations they shared. Figure 2a illustrates the social network of the patients as nodes sharing mutations. In addition, Figure 2b shows the mutations as nodes sharing patients.

**Figure 3:**
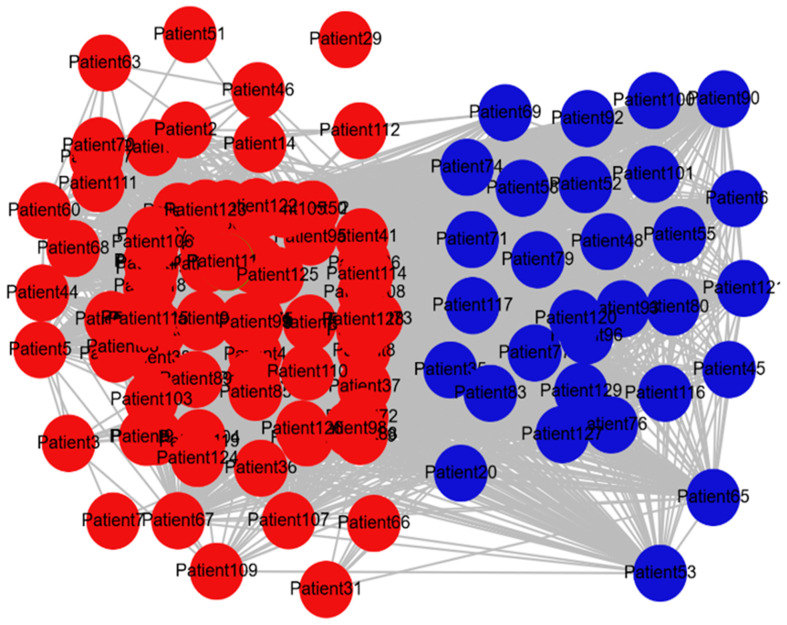
A network of patients connected through shared mutations. The thickness of the lines reflects the number of shared mutations. The colors indicate the two groups of the Louvain clustering. *Cluster 1 is represented in red and Cluster 2 is represented in blue. The patients are connected to each other by the edges of the social network (the gray lines). The lines’ thickness and number reflect the number of mutations or co-mutations linking the patients.*

The clustering process, that is, the grouping into two clusters of patients according to the mutations and co-mutations they share is illustrated in Figure 3.

Table 1 describes two clusters of patients and the main mutations that are more relevant for treatment and/or for determining the prognosis of NSCLC. The patients grouped by the algorithm into cluster 1 show a smaller number of mutations compared to cluster 2. It should be noted that this table does not show the much larger number of additional genetic abnormalities that have been found, such as copy number variations and other mutations for which there is currently no direct information regarding their relevance in the somatic context of these cancers.

The tumors in cluster 2 are expected to be more immunogenic due to a larger number of mutations and co-mutations, and therefore they are expected to show higher levels of PD-L1 and TMB compared to the tumors from cluster 1, which have a smaller number of mutations and are therefore considered immunologically “cold,” and as a result, they are expected to show lower levels of PD-L1 and TMB.

### 3.2. Patients Demographics

Table 2 shows the demographic distribution of patients diagnosed with NSCLC after grouping the patients according to the mutations they share in common. There was no statistically significant difference in terms of age, gender, and ethnicity between the two clusters of mutations.

### 3.3. Expression of Biomarkers in Patients’ Clusters

Table 3 shows the expression of PD-L1 and TMB values in the two clusters of patients according to the shared mutations. The clusters of each group of patients were formed by the social network algorithm, according to the common mutations shared by the patients. Cluster 2 patients expressed statistically significantly higher levels of both biomarkers, PD-L1 and TMB than cluster 1 patients.

**Figure 4:**
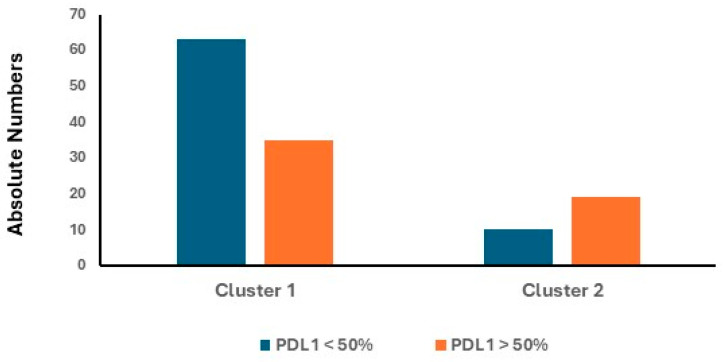
PD-L1 stratification across clusters. This graph refers to the number of patients who were found to have PD-L1 values higher or lower than the 50% cutoff point. *p* = 0.004. OR = 3.4.

In clinical settings, biological treatment against PD-L1 is administered mainly according to a cutoff point of 50% [29]. Therefore, further analysis of PD-L1 expression levels was performed. Figure 4 shows a statistically significant difference between the two clusters upon differentiating/stratifying the PD-L1 index according to this 50% cutoff point. In cluster 2, a higher rate of patients had PD-L1 values above the 50% threshold compared to patients in cluster 1. This indicates that the tumors of cluster 2 are more immunogenic compared to cluster 1 tumors.

A multivariable logistic regression analysis that included both the demographic details and the PD-L1 and TMB markers was performed in order to examine the association between the two clusters of patients according to the mutations they share (Table 4). In the multivariable analysis, the two molecular markers, PD-L1 and the TMB index are considered independent variables in predicting the two patient clusters.

## 4. Discussion

In this study, we applied a network-based approach grounded in Network Medicine principles to classify patients with non-small cell lung cancer according to shared somatic mutation profiles rather than individual driver alterations [30,31]. Using social network analysis and modularity optimization via the Louvain algorithm, we identified two biologically distinct patient clusters characterized by markedly different genomic complexity and immunologic features. An “immunogenic” cluster harboring multiple mutations (cluster 2) versus an “immune-cold” cluster (cluster 1).

To our knowledge, this is among the first studies applying patient-level social network modeling to co-mutation structure in NSCLC. The study includes multiple complex analyses; first, the patient-to-patient mutation links, second, biomarkers and combining the interactions into clusters.

The principal finding of this study is that patients grouped into the highly connected cluster (cluster 2), defined by a greater burden of co-mutations—including KRAS, TP53, STK11, and BRAF—exhibited significantly higher levels of PD-L1 expression and tumor mutational burden compared to patients in cluster 1. These findings suggest that genomic complexity, as captured by network connectivity, is associated with increased tumor immunogenicity.

This association should be interpreted with appropriate caution. The network was constructed from the count of shared somatic mutations, and TMB is itself a measure of mutational burden derived from the same sequencing assay. The higher TMB observed in the more densely connected cluster is therefore partly expected by construction rather than an independent confirmation of the clustering, and the relationship between network connectivity and TMB is not fully independent of the input data. The PD-L1 finding is less directly coupled to the network input, because PD-L1 expression was measured separately by immunohistochemistry; nevertheless, both biomarkers covary with the underlying mutational profile. Accordingly, the present results are best regarded as demonstrating internal consistency between the mutation-derived clustering and established immunogenicity markers, rather than as external validation of the clusters.

The current study findings are consistent with prior studies demonstrating that specific co-mutation patterns influence the tumor immune microenvironment [32]. In particular, pulmonary tumors harboring co-occurring mutational alterations in KRAS and TP53 have been associated with immunogenic inflamed tumor phenotypes and elevated PD-L1 expression [33], whereas combinations such as KRAS with STK11 are often linked to immunologically “cold” tumors and resistance to immune checkpoint inhibitors. Thus, the later tumors’ molecular profile presents low TMB and PD-L1 levels, indicating a lower response to immunotherapy [34].

The coexistence of these mutations within cluster 2 likely reflects a heterogeneous but overall more immunogenic genomic landscape, which may explain the higher PD-L1 and TMB levels observed.

Interestingly, although mutations such as STK11 are typically associated with reduced PD-L1 expression, their presence within a broader co-mutational context did not negate the overall immunogenic phenotype of cluster 2. This highlights an important concept: the biological behavior of tumors may depend more on combinatorial mutational patterns than on single-gene effects, supporting the rationale for integrative analytical approaches such as social network analysis [35,36].

The fact that cluster 2, which includes the STK11 mutation, had a high PD-L1 expression level, implies the dominance of co-mutation context rather than a single mutation impact such as STK11, which is usually affiliated with low PD-L1 [19]. The distinction between clusters was due solely to biological-molecular factors, and not clinical or social variables.

Another notable finding is the absence of significant differences in demographic variables between clusters. This strengthens the interpretation that the observed differences in PD-L1 expression and TMB are primarily driven by tumor-intrinsic genomic features rather than patient-related factors [34,37]. From a clinical perspective, this suggests that network-derived genomic classification [38] could provide independent and complementary information beyond standard clinical stratification.

A significant advantage of this network-based approach is the ability to characterize patients with complex co-mutation patterns that are not identified in single-mutation testing—for example, *KRAS* with *TP53* versus *KRAS* with *STK11*, which have opposing immunological profiles. Networks of this type may enable better tailoring of immunotherapy treatments in the future.

The clinical implications of these findings are potentially significant. Both PD-L1 expression and TMB are established biomarkers used to guide immunotherapy decisions in NSCLC [21,39]. The ability of a network-based model to identify patient subgroups enriched for high PD-L1 and TMB raises the possibility that such approaches could improve patient selection for immune checkpoint inhibitor therapies. Moreover, this methodology may help refine current biomarker strategies by incorporating the complexity of co-mutation patterns rather than relying on single biomarkers alone.

Luo et al. [40] stratified NSCLC patients according to somatic mutations by using metabolic-based clustering of computational biology and network propagation algorithm. That approach was validated through classification of NSCLC patient subtypes evaluated for immunotherapy followed by survival benefits.

This study has several strengths, including the use of real-world genomic and clinical data, and the application of an innovative analytical framework that captures relationships between patients rather than isolated variables. However, several limitations should be acknowledged. First, the retrospective single-center design may limit generalizability. Second, the sample size is modest, particularly for subgroup analyses. Third, the study lacks clinical outcome data, such as the response to immunotherapy or survival, which would be necessary to confirm the predictive value of the identified clusters. In addition, potential technical variability in PD-L1 assessment and TMB calculation may introduce measurement bias. Several further limitations warrant emphasis. The edge weight depended only on the number of shared gene-level alterations and did not incorporate the functional importance, pathway context, or predicted impact of individual mutations, so that driver and likely passenger alterations contributed equally to connectivity; this may influence cluster composition. Because the network and TMB were both derived from the same sequencing data, the higher TMB of the more connected cluster is partly inherent to the construction of the network and does not constitute independent validation. The proposed network-based clustering was not formally benchmarked against simpler or established alternatives—such as raw mutation count, hierarchical or Jaccard-distance clustering, or groups defined directly by KRAS, TP53, and STK11 status—so the incremental value of the social network approach over these comparators remains to be established. The smoking status and histologic subtype, although listed among the study objectives, were not consistently available in the source records and could not be incorporated into the demographic comparison or the regression model. Other potential confounders, including prior treatment, tissue source, tumor cellularity, and the timing of sampling, were likewise not adjusted for. The PD-L1 and TMB assays were not standardized across a single platform with documented inter-observer review, and the analysis was restricted to gene-level point mutations, without copy-number variation, gene expression, or epigenetic data. Finally, as a single-institution cohort drawn from one regional population, the findings may be sensitive to dataset composition and require external, multi-center validation.

Future research should focus on validating these findings in larger, multi-center cohorts and integrating clinical outcomes to assess the predictive utility of network-defined clusters. Expanding this approach to include additional genomic features—such as copy number variations, gene expression profiles, and epigenetic alterations—may further enhance its biological and clinical relevance. Furthermore, applying similar network-based methodologies across different tumor types could help to determine whether these patterns are tumor-specific or if they represent a broader principle in oncology. To establish that the social network approach adds value, future analyses should formally benchmark it against simpler comparators, including raw mutation count, hierarchical and Jaccard-distance clustering, and groups defined directly by KRAS, TP53, and STK11 status. Furthermore, they should incorporate functional weighting of mutations and a formal assessment of cluster stability. To support reproducibility, the de-identified patient-by-gene matrix, cluster assignments, biomarker values, and the analysis code and clustering settings should be made available.

## 5. Conclusions

Building a social network based on shared mutations is a feasible approach for identifying genomic subgroups in NSCLC. The cluster with multiple mutations (cluster 2) was characterized by higher levels of PD-L1 and TMB, a profile consistent with a more immunogenic phenotype. In multivariable analysis, PD-L1 and TMB were associated with cluster assignment, whereas demographic features were not. Because the cohort was retrospective and lacked immunotherapy-response and survival data, these results are hypothesis-generating: a social network-based approach may, pending prospective validation, serve as a complementary tool for characterizing co-mutation patterns relevant to immunotherapy selection.

## Figures and Tables

**Table 1:** Clusters of patients and their mutations.

Gene/Mutation	Cluster 1	Cluster 2
EGFR	2	22
ALK	2	4
ROS1	0	1
MET	2	15
RET	0	5
KRAS	0	38
BRAF	1	14
PIK3CA	2	7
TP53	4	50
STK11	0	25
KEAP1	0	1
SMARCA4	0	3
NRAS	0	1
DDR2	0	1
FGFR	0	6
PTEN	0	1
CDKN2	0	8
ARID	4	8
JAK2	1	1
Total relevant mutations	18	221

**Table 2:** Patient clusters demographics.

Variable	Cluster 1	Cluster 2	*p*-Value
Age (years), mean ± SD ^1^	72 ± 1.1	70 ± 1.1	N.S. ^1^ (>0.05)
Gender:			
Female (*n*)	39	22	N.S. ^1^ (>0.05)
Male (*n*)	35	33	
Ethnicity:			
Arab (*n*)	16	12	N.S. ^1^ (>0.05)
Jewish (*n*)	58	43	

^1^ Abbreviations: SD, standard deviation; N.S. non-significant.

**Table 3:** Biomarkers expression in co-mutation clusters of patients.

Variable	Cluster 1	Cluster 2	*p*-Value
PD-L1 ^1^	13.7 ± 2.5 ^2^	29.8 ± 4.5	0.001
TMB ^1^	5.8 ± 0.6 ^3^	7.8 ± 0.5	0.021

The values presented are mean ± SD. ^1^ Abbreviations: PD-L1, Programmed Death-Ligand 1; TMB, Tumor Mutation Burden. ^2^ The PD-L1 values in the table represent the percentage of positive staining in tumors. ^3^ TMB is presented as Mutation per megabase (Mut/Mb ± SD).

**Table 4:** Multivariable logistic regression analysis.

Variable	Betas	*p*-Values
TMB ^1^	0.107	0.032
PD-L1 ^1^	0.016	0.022
Constant	−1.231	

^1^ Abbreviations: PD-L1, Programmed Death-Ligand 1; TMB, Tumor Mutation Burden.

## Data Availability

The data that support the findings of this study are available from the Corresponding Author, [A.A.], upon reasonable request. To support reproducibility, a de-identified patient-by-gene matrix, the cluster assignments, the corresponding PD-L1 and TMB values, and the analysis code together with the network-construction and Louvain settings will be provided to qualified researchers upon reasonable request. Patient-level genomic data are not deposited in a public repository because of institutional and ethics-committee restrictions on potentially identifiable information.

## Abbreviations

The following abbreviations are used in this manuscript:

CMC	Carmel Medical Center
NSCLC	Non-Small Cell Lung Cancer
PD-1	Programmed cell death-1
PD-L1	Programmed Death-Ligand 1
SNA	Social Network Analysis
TKI	Tyrosine Kinase Inhibitor
TMB	Tumor Mutation Burden
TPS	Tumor Proportion Score
